# *In vivo* and *in vitro* protective effects of omeprazole against neuropathic pain

**DOI:** 10.1038/srep30007

**Published:** 2016-07-20

**Authors:** Sanjay K. Chanchal, Umesh B. Mahajan, Sumit Siddharth, Navyya Reddy, Sameer N. Goyal, Prakash H. Patil, Basavaraj P. Bommanahalli, Chanakya N. Kundu, Chandragouda R. Patil, Shreesh Ojha

**Affiliations:** 1 Department of Pharmacology, R. C. Patel Institute of Pharmaceutical Education and Research, Shirpur, Dist-Dhule-425405, Maharashtra, India; 2 Cancer Biology Laboratory, KIIT School of Biotechnology, KIIT University, Bhubaneswar-721024, Odisha, India; 3 Department of Pathology, Gadag Institute of Medical Sciences, Bommanahalli, Gadag-582101, Karnataka, India; 4 Department of Pharmacology and Therapeutics, College of Medicine and Health Sciences, United Arab Emirates University, Al Ain, Abu Dhabi 17666, United Arab Emirates

## Abstract

Apart from reducing the acid secretion, omeprazole inhibits activation of the nuclear factor-κB, release of inflammatory cytokines, and chemotaxis of neutrophils. These mechanisms prompted us to evaluate antineuropathic effect of omeprazole in the chronic constriction injury (CCI)-induced rat model of neuropathic pain and LPS mediated ROS-induced U-87 cells. Omeprazole at 50 mg/kg/day/oral for 14 days significantly reduced the intensity of neuropathic pain estimated as paw withdrawal latency, withdrawal pressure threshold and restored the motor nerve conduction velocity in the constricted nerve, when compared with respective groups. The histological findings revealed the protective effect of omeprazole against the CCI-induced damage. Omeprazole significantly decreased the levels of tumor necrosis factor (TNF-α), interleukin-1β (IL-1β) and interleukin-6 (IL-6) as compared to their respective control groups. It also reduced the oxidative stress by up regulating the SOD, catalase activity and decreasing MDA content. Similarly, *in-vitro study*, LPS mediated ROS-induced U-87 cells, omeprazole reduced the oxidative stress as well as the release of TNF-α, IL-1β and IL-6. Altogether, these results suggest that, neuroprotective effect of omeprazole is mediated through preventing release of proinflammatory cytokines, augmenting endogenous anti-oxidant defense system, and maintain the structural integrity of sciatic nerve from the CCI-induced structural damage and inflammatory changes.

Neuropathic pain is a debilitating condition arising from injury to the somatosensory neurons which triggers the development of allodynia and hyperalgesia^1^. Several conditions such as traumatic accidents, surgery and diseases affecting peripheral and central nervous systems contribute to etiopathogenesis of the neuropathic pain. Experimental studies reveal inflammation is the main cause of neuropathic pain. The inflammatory cytokines and oxidative stress along with allogeneic mediators released subsequent to a nerve injury induce neuropathic pain through sensitization of the nociceptive receptors^2^,^3^. The palliative treatment of neuropathic pain include the use of gabapentin, tricyclic antidepressants, α2-δ calcium channel ligands, slow serotonin reuptake inhibitors and local aesthetics^4^. Gabapentin is recommended as the standard of care in the neuropathic pain^5^. However, the absorption of gabapentin follows saturation kinetics upon oral administration and even at higher doses its bioavailability does not improve. This renders a need for dose-titration to achieve the expected therapeutic effects^6^. Other antineuropathic drugs including anticonvulsants, tricyclic antidepressants and opioids have a limited effect and possess abuse liabilities or intolerable side-effects. Thus, there is a continuous demand for novel antineuropathic drugs which could be more safe and effective. The recent approach of repositioning of therapeutic agents for discrete indications may prove to be useful in the exploration of certain neuroprotective, anti-inflammatory and antioxidant agents to be added as an add-on therapy to the primary recommended therapy for neuropathy and associated pain.

In preview of such repositioning approach, many recent preclinical and clinical evaluations have highlighted diverse effects of omeprazole other than its proton pump inhibitory actions. Omeprazole is a proton pump inhibitor that is widely used in the treatment of peptic ulcer^7^. *In vitro* and and *in vivo* evaluations have proved omeprazole have carbonic anhydrase inhibitory^8^, anti-inflammatory^9^ and antioxidant^10^ activities. Omeprazole following *in vitro* co-incubation with isolated human neutrophils showed to reduce hemotaxis and oxidative radical production induced by bacterial chemotactic tetrapeptide, N-formyl-Met-Leu-Phe (fMLP)^11^. Additionally, omeprazole also antagonizes the inflammatory response of mouse macrophages infected with *salmonella enterica*. In this *in vitro* study, it delayed the IκB degradation, blocked nitric oxide production and reduced the secretion of proinflammatory cytokines from the infected macrophages^12^. The anti-inflammatory activity of omeprazole and other proton pump inhibitors (PPIs) in a murine model of asthma through IL-4 and IL-13 signalling STAT-6 (signal transducer and activator of transcription-6) activation were also reported^13^. On the other hand, omeprazole also reduces the LPS-induced release of TNF-α and IL-6 from the human microglial cell culture and human monocytes culture *in vitro*. This effect is proposed to contribute to the neuroprotective effect of omeprazole against the microglial and monocytic toxicity^14^. Further, it has also been revealed to reduce the IFN-γ induced astrocyte toxicity and phosphorylation of STAT3^14^. Moreover, it also inhibits the carrageenan-induced acute paw inflammation in rats^10^,^15^. Considering these findings, we hypothesized that omeprazole should reduce the post-injury nerve damage and inhibit the development of nerve injury-related neuroinflammation and neuropathic pain. The present study was designed to investigate the effects of chronic oral administration of omeprazole employing the CCI-induced neuropathic pain in rats.

## Results

### Effect of omeprazole on cold, warm & mechanical allodynia in CCI-induced neuropathic pain

**Figure 1:**
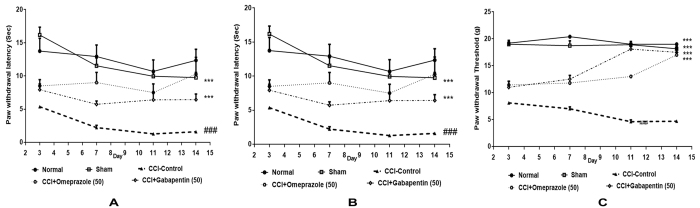
Omeprazole ameliorated the cold, warm and mechanical stimuli induced allodynia in CCI induced neuropathic pain in rats. (**A**) In cold allodynia treatment with omeprazole (50 mg/kg p.o. for 14 days) showed time dependent increase in the paw withdrawal latency in CCI control rats without influencing the measures in sham rats. (**B**) In warm allodynia treatment with omeprazole (50 mg/kg p.o. for 14 days) showed time dependent increase in the paw withdrawal latency and threshold in CCI-control rats but not in sham rats. (**C**) In Mechanical allodynia, treatment with omeprazole (50 mg/kg p.o. for 14 days) showed time dependent increase in the paw withdrawal latency or threshold in CCI-control rats but not in sham rats. Data are expressed as the mean ± S.E.M. (n = 8/group). Significance was determined by repeated measures ANOVA followed by the Dennett’s post-hoc test. The statistical significance of difference of CCI-control compared to sham was shown as ^###^*p* < 0.001, whereas the statistical significance of difference in the omeprazole and gabapentin treated groups as compared to CCI-control was designated as ***p < 0.001.

Figure 1A–C illustrates the effects of omeprazole on cold, warm & mechanical allodynia during CCI-induced neuropathic pain in rats. In the plantar test to determine cold allodynia, the paw withdrawal latency (PWL) was significantly increased as compared to sham group (14.4 ± 1.17 sec Vs. 3.86 ± 1.16 sec, P < 0.001). Oral treatment with omeprazole (50 mg/kg/day) for 14 days significantly increased the PWL as compared to the CCI-control animals [9.37 ± 0.56 sec Vs. 3.86 ± 1.16 sec; F (117.3) DF = 4, P < 0.001]. Similar effect was observed in GP treated group when compared with their respective control group (7.62 ± 0.34 sec Vs. 3.86 ± 1.16 sec; P < 0.001; Fig. 1A).

Figure 1B shows the effect of omeprazole on warm allodynia after CCI surgery. The baseline level of PWL of the CCI-induced hind paw was 2.610 ± 0.94 sec, when challenged with warm water, whereas the sham operated rats didn’t show any change in the warm allodynia. Omeprazole (50 mg/kg/day/oral) for 14 days treatment significantly increased the PWL as compared to the CCI-control group (8.82 ± 0.57 sec Vs. 2.610 ± 0.94 sec; [F (42.74) DF = 4, P < 0.001]).

The paw withdrawal pressure threshold (PWT) was estimated using the electronic von Frey apparatus. The CCI-control group shows significant decreased in PWT as compared to sham operated animals (6.103 ± 0.86 g Vs. 18.7 ± 0.19 g, P < 0.001, Fig. 1C). The omeprazole treatment significantly increased the PWT as compared to respective control group (13.31 ± 1.27 g Vs. 6.103 ± 0.86 g; [F(17.23) DF = 4, P < 0.001]).

### Effect of omeprazole on motor nerve conduction velocity in CCI-induced neuropathic pain

**Figure 2:**
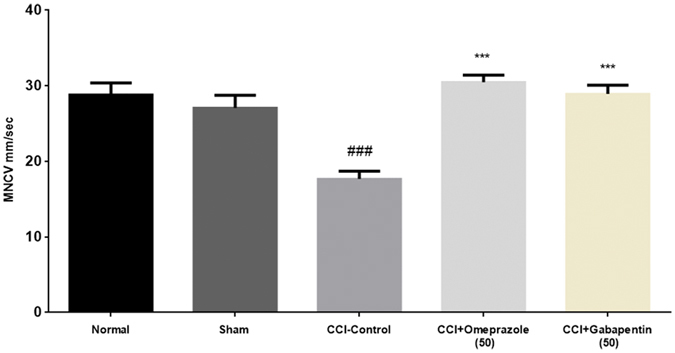
Omeprazole alleviate the MNCV in CCI-induced neuropathic pain in rats. Administration of omeprazole (50 mg/kg p.o. for 14 days) for CCI rats showed significant improvement in the MNCV. Data are expressed as the mean ± S.E.M. (n = 8/group). Significance was determined by two way ANOVA followed by the Bonferroni’s multiple comparison test. The statistical significance of difference of CCI-control compared to sham was shown as ^###^*p* < 0.001, whereas the statistical significance of difference in the omeprazole and gabapentin treated groups as compared to CCI-control was designated as ***p < 0.001.

MNCV was evaluated on the day 14^th^ after the respective treatments. There was a significant reduction (17.68 ± 1.01 mm/sec; P < 0.001) in the MNCV in the CCI-control group. The rats treated with omeprazole 50 mg/kg/day/oral for 14 days showed significant increase (30.50 ± 0.91 mm/sec) in the MNCV as compared to the CCI-control group [F (15.62) DF = 4, P < 0.001]. It is noteworthy that the alleviative effect of omeprazole on the MNCV was comparable to the gabapentin (Fig. 2).

### Effect of omeprazole on biochemical parameters in CCI-induced neuropathic pain

**Figure 3:**
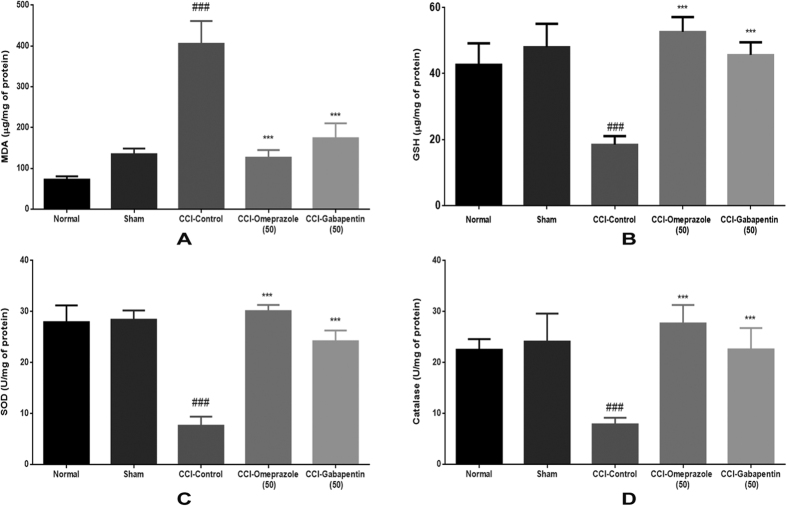
Omeprazole reduces the oxidative stress in CCI-induced neuropathic pain in rats. CCI surgery in rats induces distinct oxidative stress, while treatment of omeprazole (50 mg/kg p.o. for 14 days) alleviates the increase in oxidative stress. (**A**) Malondialdehyde content (MDA) (**B**) Reduced glutathione (GSH). (**C**) Superoxide dismutase (SOD). (**D**) Catalase. Each value represents the mean of 5–8 experiments ± S.E.M. Data are expressed as the mean ± S.E.M. (n = 8/group). Significance was determined by one-way ANOVA followed by Dennett’s post-hoc test. The statistical significance of difference of CCI-control compared to sham was shown as ^###^*p* < 0.001, whereas the statistical significance of difference in the omeprazole and gabapentin treated groups as compared to CCI-control was designated as ***p < 0.001.

Oxidative stress status was evaluated by the measurement of MDA as a marker of lipid peroxidation, GSH as well as SOD and catalase as markers of enzymatic and non-enzymatic antioxidant defence systems. CCI induced a state of marked oxidative stress as demonstrated by a significant increase (405.4 ± 55.97 μg/mg of protein) in the MDA level and a significant decrease in the GSH (18.45 ± 2.59 μg/mg of protein), activity of SOD (7.613 ± 1.77 U/mg of protein) and catalase (7.84 ± 1.32 U/mg of protein) levels in sciatic nerve homogenates as compared to control group. The treatment with omeprazole 50 mg/kg/day/oral for 14 days alleviated the effect CCI-induced oxidative stress on the levels of MDA, GSH. It also had significant restorative effect on the activities of SOD and catalase enzymes as compared to CCI-control group (Fig. 3).

### Effect of omeprazole on proinflammatory cytokines in CCI-induced neuropathic pain

**Figure 4:**
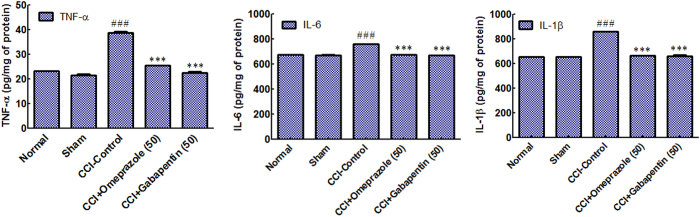
Omeprazole condenses the release of pro-inflammatory cytokines in CCI-induced neuropathic pain in rats. (**A**) Tumour necrosis factor-α (TNF-α). (**B**) Interleukin-6 (IL-6). (**C**) Interleukin-1β (IL-1β). Data are expressed as the mean ± S.E.M. (n = 8/group). Significance was determined by one-way ANOVA followed by Bonferroni’s multiple comparison test. The statistical significance of difference of CCI-control compared to normal group was shown as ^###^*p* < 0.001, whereas the statistical significance of difference in the omeprazole and gabapentin treated groups as compared to CCI-control was designated as **p < 0.005 and ***p < 0.001.

To further define the mechanisms by which cytokine signalling may be associated with CCI induced neuropathic pain, we examined the effect of omeprazole on release of pro-inflammatory cytokine expression of TNF-α, IL-1β and IL-6 in the nerve tissue homogenate. A significant increase in the cytokine levels in the CCI-control group (TNF-α [38.8 ± 1 Vs. 23.2 ± 0.9 pg/mg of protein respectively]), IL-6 [761.7 ± 1.8 Vs. 675.5 ± 0.7 pg/mg of protein respectively] and IL-1β [859 ± 0.7 Vs. 656.4 ± 1.2 pg/mg of protein respectively]) compared to normal group. The groups treated with omeprazole (50 mg/kg/day/oral) for 14 days showed a significant reduction in these elevated cytokines (TNF-α [25.5 ± 0.8 Vs. 23.2 ± 0.9 pg/mg of protein respectively], IL-6 [676.2 ± 1.0 Vs. 656.4 ± 1.2 pg/mg of protein respectively] and IL-1β [666.5 ± 1.1 Vs. 656.4 ± 1.2 pg/mg of protein respectively]) almost to the normal levels indicating that omeprazole exerts a consistent inhibitory effect on the cytokine release (Fig. 4).

### Effect of omeprazole on tissue architecture of sciatic nerve in CCI-induced neuropathic pain

**Figure 5:**
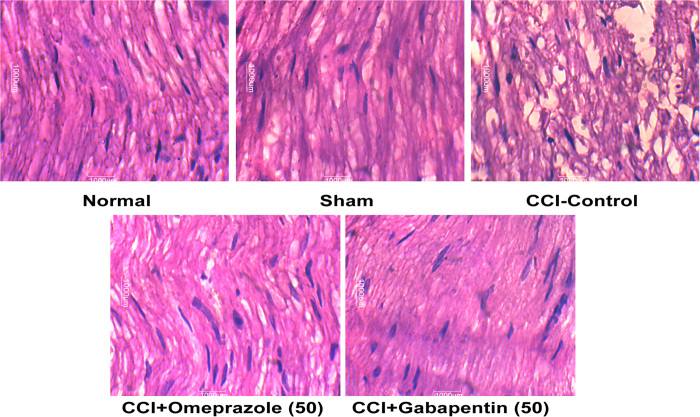
Omeprazole prevents histological variations in CCI-induced neuropathic pain (Hematoxylin and eosin X100). The normal rat’s sciatic nerve section showed normal structure, architecture and no inflammation. Sham operated rat’s showed same structure as observed in the normal rats, not any marked changes were observed. CCI-control animals showed swelling of nerve fibers, derangement and showed increase in the inflammatory cell infiltration as well as pronounced inflammation. Omeprazole treated rats showed decrease in the swelling as observed by decrease in inflammation. Gabapentin treatment with rats normalised the derangement observed in the CCI rats. Scale bar 1000 μm.

The CCI induced a noticeable histological perturbation as revealed in the longitudinal nerve sections including axonal swelling, neutrophil migration, and an increase in the number of Schwann satellite cells and derangement in the nerve architecture. Omeprazole 50 mg/kg/day/oral for 14 days treatment protected the sciatic nerve from the CCI-induced structural damage and inflammatory changes (Fig. 5; Table 1).

### Effect of omeprazole on LPS-induced oxidative stress in primary glioblastoma U-87cells

**Figure 6:**
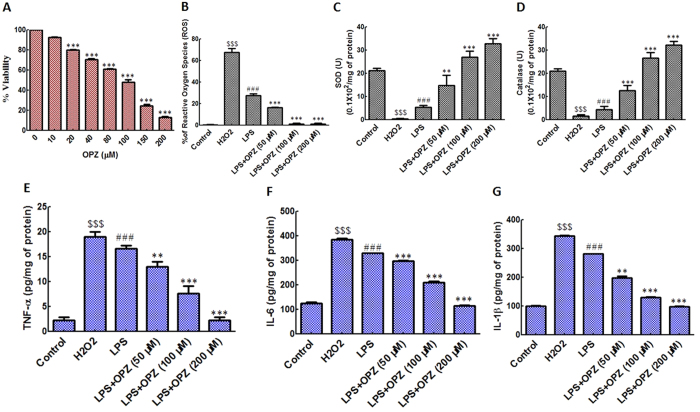
Effect of omeprazole on LPS mediated ROS, SOD, catalase and cytokines in U-87 cells. (**A**) MTT cell viability assay after omeprazole (OPZ) exposure in U-87 cells. The significance of difference in the treatment groups compared to untreated control was designated as ***p < 0.001. (**B**) Various concentrations of omeprazole were treated in LPS pre-treated (500 ng/ml for 20 min) cells prior to measure the ROS. Graphical representation of ROS positive population analyzed flow cytometrically after DCFH-DA staining. (**C**) Biochemical detection of SOD, and (**D**) Catalase after omeprazole treatment in LPS pre-treated U-87 cells. (**E**), (**F**) and (**G**) were the expressions of TNF-α, IL-6 and IL-1β , respectively, after omeprazole treatment in LPS pre-treated cells measured by indirect ELISA. H_2_O_2_ is used as a positive control for ROS. LPS indicate U-87 cells treated with LPS alone. LPS + OPZ represents indicated concentration of Omeprazole treatment on LPS pre-treated U-87 cells. Data represents mean ± S.E.M. of 8 independent experiments. The significance of difference of H_2_O_2_ (positive control of ROS in *in-vitro* experiments) compared to control was designated as ^$$$^p < 0.001. The statistical significance of difference of LPS group compared to control group was shown as ^###^*p* < 0.001, whereas the statistical significance of difference in the treatment groups as compared to LPS group was designated as **p < 0.005 and ***p < 0.001.

To study the impact of omeprazole *in vitro*, we first induced ROS by LPS treatment (500 ng/ml for 20 min) in primary glioblastoma U-87cells. After LPS stimulation, we treated the cells with graded concentrations of omeprazole and carried out a series of cell based assays. To check the anti-cell proliferative activity of omeprazole on LPS-induced cells, an MTT cell viability assay was performed. Figure 6A revealed that omeprazole decreased LPS-mediated ROS induced cell viability dose dependently with an IC_50_ of 100 μM. However, no appreciable cytotxicity was observed when cells were treated with omeprazole (0–200 μM) without LPS stimulation (data not shown). Interestingly, no cytotoxicity was also noted after treatment of LPS alone for 20 min (data not shown). Flow cytometric analysis of ROS production by DCFH-DA staining revealed a 25% ROS positive population after LPS induction which reduced to 15% after 50 μM of omeprazole treatment. Complete diminished (less than1%) ROS positive population was noted at 100 μM omeprazole exposure in LPS pre-treated cells (Fig. 6B). H_2_O_2_ is used as positive control for ROS generation. LPS treated cells displayed reduced activities of SOD and catalase which was alleviated after omeprazole exposure (Fig. 6C,D). Omeprazole alleviated the SOD and catalase even more than basal level at 100 μM (Fig. 6C,D).

Next, we measured the cytokine profile (TNF-α, IL-6 and IL-1β) after omeprazole exposure in LPS pre-treated cells. LPS increased cytokines level compared to untreated cells. Interestingly, omeprazole dose dependently decreased the LPS-induced cytokines level in cells. Omeprazole treatment almost brings down the cytokines level to basal level at 200 Μm (Fig. 6E–G).

## Discussion

The present study demonstrated that in addition to its PPI effect, omeprazole exhibits a protective effect by improving nerve conduction velocity and PWL; restoring the endogenous antioxidant system; preventing the increased level of inflammatory cytokines such as TNF-α, IL-1β and IL-6; reducing lipid peroxide metabolism; and preserving the structure and morphology of sciatic nerve via inhibiting proinflammatory cytokines. The present study shows for the first time that omeprazole exerts its neuroprotective effect not only through its antioxidant effect but also in part by inhibiting cytokines release. Therefore, our study provides direct evidence that the cytokines signaling pathway plays a key role in regulating oxidative stress and subsequent nerve injury in CCI-induced neuropathic pain. To the best of our knowledge, this is the first study to describe the neuroprotective effect of omeprazole through inhibiting cytokines release in CCI-induced model of neuropathic pain in rats.

Several other drugs like minocycline^16^ and atorvastatin^3^ are reported to attenuate the CCI-induced neuropathic pain through anti-inflammatory and antioxidant actions. Omeprazole is previously reported as an anti-inflammatory^13^,^17^, antioxidant^18^, carbonic anhydrase and cytokine release inhibitor^8^,^19^, and neuroprotective agent^14^. Recently, there is an increased interest in the repositioning of the proton pump inhibitors as anticancer agents through targeting the thioesterase domain of the fatty acid synthase^20^. In light of these preclinical studies, we evaluated omeprazole for its antineuropathic efficacy in the CCI induced neuropathy model in rats and ROS induced model of U-87 human neuronal cell line. We selected the oral dose of 50 mg/kg of omeprazole for evaluation of the antineuropathic efficacy. At this dose it significantly inhibits the carrageenan induced acute paw inflammation. Such efficacy of omeprazole is reported earlier by El-Nezhawy^10^. The oral dose of 50 mg/kg/day of gabapentin was similarly selected dependent upon a prior report on its antineuropathic effect in CCI rat model^21^. CCI of the sciatic nerve in rats is the most commonly used model to induce neuropathic pain in rats and it mimics the pathophysiology of neuropathic pain in human^22^. In CCI model, the loose ligation compresses the nerve fibres and induces nerve damage, a consequent release of various inflammatory mediators from the mast cells, neutrophils and macrophage. CCI induces a persistent neuropathic pain state characterized by spontaneous pain, allodynia and hyperalgesia^3^,^22^. The causative factors of neuroinflammation induced by CCI include pro-inflammatory cytokines, prostaglandins, and reactive oxygen species. In addition to the inflammatory mediators, an increased concentration of extracellular H^ + ^ions at the site of injury lowers the pH of the extracellular fluid. This acidic pH activates the TRPV1 (transient receptor potential vanilloid receptor 1- an acid sensitive ion channel) which serves as a sole sensor for the protons^23^. Activated TRPV1 alters calcium levels in the sensory neurons and sensitizes the capsaicin-sensitive neurons^24^. The peripheral injury induced by CCI also leads to the release of tachykinins and neurotransmitters such as glutamate, calcitonin gene-related peptide, and γ-amino butyric acid. Prolonged release and binding of these substances to neural receptors activates the *N*-methyl-D-aspartate receptors, which causes an increase in the intracellular calcium levels^25^. Increased intracellular calcium through N-type calcium channels in the central nervous system plays an important role in the maintenance of the pain sensation in the central nervous system^26^.

The other remarkable observation obtained in the present study was the ability of omeprazole to reduce the cytokine levels in CCI injury in rats. The pathogenesis of CCI-induced neuropathic pain and hyperalgesia involves a role of the immune cells that infiltrate the damaged nerve^27^ and the inflammatory immune mediators like IL-6^28^, IL-1β^29^ and TNF-α^30^. Damage to the nerve activates neurons and glial cells and releases pro inflammatory mediators like TNF-α, IL-1β and IL-6. In the present study, it was found that CCI control animals showed a significant rise in the levels of these cytokines, whereas, omeprazole significantly decreased the increased levels of cytokines. Other studies suggesting that omeprazole possesses anti-inflammatory activity, reported to reduce vascular permeability and experimentally induced colitis by suppressing elevated level of inflammatory mediators such as neutrophils IL-1β, TNF-α and IFN-γ^14^,^28^. An *in vitro* investigation on the stimulated mouse macrophages has shown that omeprazole reduces the release of inflammatory cytokines. Omeprazole and other proton pump inhibitors are also known to inhibit IL-4 and IL-13 signalling STAT6 activation and thereby exert anti-inflammatory effects^13^. LPS induced ROS in U-87 human glioma cells which allevated pro-inflammatory cytokines (TNF-α, IL-6 and IL-1β). Omeprazole suppressed the elevated inflammatory cytokines, restoring the normal profile which revealed antineuropathic efficacy of omeprazole via reduction of ROS *in vitro*. Hence, the antineuropathic efficacy of omeprazole may involve its inhibitory effects on the release of these inflammatory cytokines. Omeprazole reduces the chemotaxis of neutrophils^11^, which also might contribute to the decreased amounts of cytokines in the nerve homogenates. Omeprazole is also known to protect the astrocytes from IFN-γ induced toxicity. *In vitro* study has proved its neuroprotective effects against monocytic and microglial damage^14^. In addition to reduction in chemotaxis of neutrophils, omeprazole reduces the oxidative stress through scavenging hydroxyl radicals and also inhibits the oxidative stress induced DNA damage related apoptosis^18^. The CCI-induced ischemic hypoxia causes disturbance in the secondary metabolites in the afflicted nerve fibres and induces oxidative stress. Further, the decreased nerve energy and degeneration of the nerve fibres due to neuronal ischemia reduces the MNCV^31^,^32^. In present study, omeprazole inhibited the decrease in the MNCV. It was observed in present study, that there was a significant increase in the PWT and PWL in the mechanical and thermal allodynia tests in omeprazole treated rats. This effect may be attributed to the antioxidant and anti-inflammatory property of omeprazole. Omeprazole reduced the oxidative stress induced by CCI and this was evident from the reduced malondialdehyde and restoration of the depleted GSH, catalase and SOD. Thus, the antioxidative effect of omeprazole may be considered as one of the mechanisms of its antineuropathic effect.

Considering the time tested safety profile of omeprazole and its antineuropathic efficacy observed in this study, it is suggested that omeprazole may be considered for further evaluation in preclinical and clinical studies against painful neuropathic conditions. Further investigation in this direction may lead to the repositioning of omeprazole in treatment of inflammatory and painful disease conditions. Whether the present conclusions can be extrapolated to a clinical scenario remains to be determined in clinical studies.

## Methods

### Animals

Adult male Wistar rats (180–250 g) were procured from the laboratory animal facility of our Institute. They were housed in standard temperature/humidity conditions and environment (12 h light/dark cycle). All animals were provided standard pellet diet and water *ad libitum* all time except during the estimation of the behavioural parameters. The animals were maintained in conformity with the regulations laid down by the Committee for the Purpose of Control and Supervision of the Experiments on Animals (CPCSEA) constituted under the Prevention of the cruelty to animals Act, 1960, Ministry of Environment and Forests, Government of India. The experimental protocols were approved by the Institutional Animal Ethics Committee of R. C. Patel Institute of Pharmaceutical Education and Research, Shirpur, Dist-Dhule, Maharashtra, India (Protocol approval # IAEC/RCPIPER/2014-15/09). All the tests complied with the recommendations of the International Association for the Study of Pain.

### Chemicals

Omeprazole was obtained from Pharmachem R&D Laboratories, India. Gabapentin was gifted by Mylan laboratories, India. Cytokine ELISA Ready SET-Go kits for mouse IL-1β (Cat: 887013-22; Batch No. E09323-1645), IL-6 (Cat: 837064-22: Batch No. E09358-1645) and TNF-α (Cat: 837324-22: Batch No. E09479-1645) were procured from e-Biosciences Incorporation, USA. Freshly prepared drug suspensions in 0.5% carboxymethyl cellulose were used for oral administration to rats. LPS (Cat: L2880; Lot No. 025M4040V) was purchased from Sigma-Aldrich, St. Louis, Missouri, USA.

### Induction of CCI in rats

The CCI surgery was performed as described by Aswar *et al*.^22^. The rats were anaesthetized under pentobarbital sodium (60 mg/kg, intraperitoneal). The common sciatic nerve of the right hind limb was exposed at the middle of the thigh by a blunt dissection through biceps femoris. Proximal to the trifurcation of sciatic nerve, about 5–7 mm of the nerve was freed off the adhering tissue, and four loose ligatures (4.0 silk) were put around it approximately 1 mm apart. After performing nerve ligation, muscular and skin layers were immediately sutured and povidone-iodine solution was applied externally. After the surgery, the rats were kept in individual cages and were allowed to recover. The respective drugs treatments were initiated on the next day after the surgery.

### Cell culture and treatment

The human glioma cell line; U-87 was grown and cultured in DMEM containing 10% FBS, 100 U/ml penicillin, 100 μg/ml of streptomycin, 1.5 mM L-Glutamine in a humidified atmosphere of 5% CO_2_ at 37 °C. After confirming that the cells attained 80% confluence, the media was replaced with fresh media containing 500 ng/ml LPS for 20 min for ROS induction. Various concentrations of omeprazole were added in LPS pre-treated cells for another 24 h prior to perform other experiments. A fixed concentration (10 μM) of H_2_O_2_ was treated for 30 min to produce ROS and used as positive control.

### MTT cell viability assay

To check the cytotoxicity of omeprazole in LPS mediated ROS-induced U-87 cells, we have performed an MTT cell viability assay according to the protocol referred earlier^33^. Briefly, 8000–10,000 cells were seeded in triplicate in 96 well plate and grown to 80% confluence. Then, cells were treated with 500 ng/ml LPS for 20 min for ROS induction. LPS containing media was aspirated and the cells were further treated with a varied concentration of omeprazole for another 24 h. Then, 0.05% MTT reagent was added to each well and incubated overnight for the formation of formazan crystal. The colour intensity was spectrophotometrically (Berthold, Germany) measured at 570 nm after dissolving the formazan crystals in DMSO. Data was calculated and represented as percent viability against omeprazole concentrations.

### Experimental design

The anti-inflammatory activity of orally administered omeprazole at 10, 30, and 50 mg/kg dose was measured in rat model of carrageenan-induced paw edema (data not shown). At the dose of 50 mg/kg, omeprazole significantly inhibited the induction of carrageenan-induced paw edema. Therefore, the dose of 50 mg/kg was chosen further to investigate the efficacy of omeprazole against CCI induced neuropathy and was compared with well reported anti-neuropathic dose of gabapentin (50 mg/kg p.o.).

After CCI induction, the rats were allowed to habituate for 3 days. Treatment of omeprazole was initiated on the next day of CCI surgery. The thermal and mechanical allodynia were measured as described by Demir^2^ on 3^rd^, 7^th^, 11^th^ and 14^th^ days after surgery. The paw withdrawal latency (PWL) was observed with a maximum cut off time of 20 sec. For the determination of the thermal allodynia, the right paw of each rat up to the ankle joint was immersed in warm water (40 ± 1 °C) and cold water (12 ± 1 °C). The mechanical allodynia was measured using electronic Von-Frey apparatus using super-tips probes (2390 series, IITC Life Sciences Incorporation) and paw withdrawal threshold (PWT) was observed with cut-off pressure at 30 gm.

On the 14^th^ day post-surgery, the rats were anesthetized with pentobarbital sodium (60 mg/kg, intraperitoneal) in the temperature controlled atmosphere (25 °C). Sciatic-tibial motor MNCV was measured by stimulating proximally at the sciatic notch and distally at the knee via bipolar needle electrodes (Power Lab/ML856; AD Instruments, Australia; frequency 0.10 Hz, duration 0.1 ms, amplitude 1.5 V). After single stimulus the compound muscle action potential was recorded from the first interosseous muscle of the hind-paw by unipolar pin electrodes. The recording was a typical biphasic response with an initial M-wave which is a direct motor response due to stimulation of motor fibers. The MNCV was calculated as the ratio of the distance (mm) between both sites of stimulation divided by the difference between proximal and distal latencies measured in ms^31^.

After recording of MNCV, the rats were sacrificed using overdose of pentobarbital sodium (intraperitoneal) and the injured right sciatic nerve was isolated along with 1 cm segments on proximal and distal side of the CCI injury. A 5 mm central portion of the isolated nerve segment was further processed for histological examination. The sections of 4 μm thickness were obtained and were stained with haematoxylin and eosin. The stained sections were examined under the light microscope for structural alterations including fibre derangement, swelling of nerve fibre and presence of activated satellite cells and Schwann cells.

A 10% homogenate of the remaining segments of sciatic nerve from each rat was prepared in ice chilled phosphate buffer (50 mM, pH 7.4). The homogenate was centrifuged at 2000 g for 20 min at 4 °C and the aliquots of the supernatant were used to estimate the content of lipid peroxidation measures as malondialdehyde (MDA), reduced glutathione (GSH), catalase, and superoxide dismutase (SOD) as follows.

Lipid peroxidation in the nerve tissue was determined by measuring MDA content as described by Ohkawa *et al*.^34^. Briefly, 0.2 ml of the tissue homogenate was mixed with 0.2 ml of 8.1% sodium dodecyl sulphate, 1.5 ml of 30% acetic acid (pH 3.5) and 1.5 ml of 0.8% thiobarbituric acid. The reaction mixture was heated for 60 min at 95 °C and then cooled on ice. After cooling, 1.0 ml of distilled water and 5.0 ml of n-butanol: pyridine (15:1 v/v) solution were added and centrifuged at 5000 rpm for 20 min. The absorbance of the generated pink colour in organic layer was measured at 532 nm. The reagent; 1,1,3,3-tetraethoxypropane (Sigma Chemicals, USA) was used as the standard MDA and the levels were expressed as μg/mg of protein.

The neuronal GSH was estimated by the method of Moron *et al*.^35^. Briefly, 100 μl of tissue homogenate was mixed with 100 μl of 10% trichloro acetic acid and vortexed. The contents were then centrifuged at 5000 rpm for 10 min. Subsequently 0.05 ml of supernatant was mixed with a reaction mixture containing 3.0 ml 0.3 M phosphate buffer (pH 8.4) and 0.5 ml of DTNB. Within 10 min, the absorbance was measured spectrophotometrically at 412 nm. The concentration of GSH was determined from a standard curve produced using commercially available standard GSH (Sigma Chemicals, USA). The levels of GSH were expressed as μg/mg of protein.

Catalase activity was estimated by the method described by Aebi^36^. Briefly, to 50 μl of tissue supernatant a cocktail of 1.0 ml of 50 mM phosphate buffer (pH 7) and 0.1 ml of 30 mM hydrogen peroxide was added. The absorbance which read as reduction in optical density was measured spectrophotometrically on every 5 sec for 30 sec at 240 nm. The activity of catalase was expressed as U/mg protein.

SOD activity was determined by the method described by Marklund and Marklund^37^. Briefly, to 25 μl of tissue supernatant a cocktail of 100 μl of 500 mM Na_2_CO_3_, 100 μl of 1 mM EDTA, 100 μl of 240 μM/ NBT, 640 μl of distilled water, 10 μl of 0.3% Triton x 100 and 25 μl of 10 mM Hydroxylamine was added. The readings were recorded spectrophotometrically in kinetic mode at interval of 1 min up to 3 min at 560 nm. The enzyme activity was expressed as U/mg protein.

As the IC_50_ of omeprazole treated LPS stimulated U-87 cells was 100 μM with no detectable cytotoxicity on the non-induced cells, so, for other *in-vitro* experimentation, LPS mediated ROS induced U-87 cells were treated with 50, 100 and 200 μM of omeprazole. Total cellular lysate was prepared using modified RIPA lysis buffer and 50 μg of protein was used to determine the activity of SOD and catalase. The quantification of TNF- α, IL-1β and IL-6 was performed both in homogenate and cell culture supernatant using 50 μg of protein by the commercially procured ELISA kits^37^.

### Statistical analysis

The statistical analysis was performed using Graph Pad Prism version 6.0 software, USA. The results are expressed as mean ± S.E.M. The data from the behavioural parameters were analysed by repeated measure one-way analysis of variance (ANOVA). The data sets of the biochemical parameters are analysed using one-way ANOVA followed by Dennett’s post-hoc test and Bonferroni’s multiple comparison test for cytokine analysis. The results are expressed as F (DFn, DFd). The statistical significance of difference in the central tendencies of treatment groups as compared to CCI group was designated as **p < 0.005 and ***p < 0.001. Whereas, the significance of difference of CCI and LPS group compared to control group was shown as ^###^*p* < 0.001. The significance of difference of H_2_O_2_ (positive control of ROS in *in-vitro* experiments) compared to control was designated as ^$$$^*p* < 0.001.

## Additional Information

**How to cite this article**: Chanchal S. K. *et al. In vivo* and *in vitro* protective effects of omeprazole against neuropathic pain. *Sci. Rep.* **6**, 30007; doi: 10.1038/srep30007 (2016).

## Figures and Tables

**Table 1:** Effect of omeprazole on tissue architecture of sciatic nerve in CCI-induced neuropathic pain.

Groups	Fiber derangement	Swelling of nerve fiber	Satellite cells and Schwann cells
Normal	−	−	−
Sham	−	−	−
CCI-Control	++	++	++
CCI+Omeprazole (50)	+	+	−
CCI+Gabapentin (50)	−	−	−

(−) Nil, (++) Severe, (+) Mild.
